# Genomic Insights Into the Body Size Evolution in Felidae (Mammalia: Carnivora)

**DOI:** 10.1002/ece3.74405

**Published:** 2026-09-22

**Authors:** Shihu Zhao, Tian Xia, Xuesong Mei, Chen Qiu, Ying Zhang, Chao Zhao, Xiaoyang Wu, Xiaodong Gao, Jiaohui Fang, Jianqun Ding, Zhenglong Wang, Xiufeng Yang, Honghai Zhang

**Affiliations:** ^1^ School of Life Sciences Qufu Normal University Qufu Shandong Province China; ^2^ Shandong Key Laboratory of Wetland Ecology and Biodiversity Conservation in the Lower Yellow River Qufu Normal University Qufu Shandong Province China; ^3^ School of Pharmaceutical and Food Engineering Zibo Polytechnic University Zibo Shandong Province China; ^4^ Jining Nansihu Nature Reserve Service Center Jining Shandong Province China; ^5^ Forestry Protection and Development Center of Tai'an City Tai'an Shandong Province China

## Abstract

Body size represents one of the most fundamental adaptive traits in mammals, but the genetic architecture underlying the extensive body size diversification observed in the Felidae family remains largely uncharacterized. Here, we present the first comprehensive comparative genomic analysis of Felidae body size evolution using high‐quality reference genomes from 17 species, including two newly reannotated genomes. Using phylogenetic generalized least squares (PGLS) analysis, we identified 35 body‐size‐associated genes (BAGs), most of which are functionally implicated in skeletal development, thyroid hormone synthesis, and lipid metabolism. Functional enrichment analysis revealed that positively selected genes (PSGs) in large‐bodied Felidae were predominantly enriched in DNA repair pathways, whereas PSGs in small‐bodied Felidae were primarily involved in energy metabolism and lipid metabolism processes. In summary, the adaptive evolution of both core signaling pathways and key functional genes has jointly driven body size diversification in Felidae. This study uncovers new molecular insights into how mammalian body size evolves, while also generating a valuable genomic dataset for subsequent functional research.

## Graphical abstract

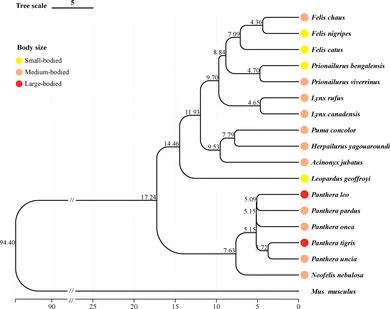

## 1 Introduction

Body size is one of the most intuitive and fundamental traits of organisms. It is closely linked to multiple aspects of a species' biology—including life history, ecological niche, and extinction risk—and reflects its adaptation to the environment (Feldman et al. 2016; Slavenko et al. 2016). Consequently, the evolution of body size has long been a central focus of research in evolutionary biology. Most animal species on Earth tend toward relatively small body sizes, a pattern widespread across major vertebrate lineages including mammals, birds, amphibians, and reptiles (Stanley 1973; Kozłowski and Gawelczyk 2002; Clauset and Erwin 2008). Regarding evolutionary trends in mammalian body size, the academic community holds a variety of differing viewpoints. One study first proposed that Cenozoic mammals exhibited an overall evolutionary trend toward increasing body size, a pattern known as Cope's rule (Cope 1887). Another study suggests that the body size distribution pattern in mammals is influenced by multiple factors: species generally evolve toward larger sizes, a minimum size threshold exists, and larger species face a higher risk of extinction (Clauset and Erwin 2008). Additionally, some research indicates that mammals possess a single, natural optimal body size (Stanley 1973; McKinney 1986). However, another investigation proposed that mammalian body‐size evolution involves three optima—two stable equilibria at large and small sizes, respectively, and one unstable equilibrium at a medium size (Alroy 1998; Finarelli 2007). This explains why medium‐sized mammal species are relatively scarce in nature, whereas large and small species are more numerous.

Felidae belongs to the order Carnivora within the class Mammalia. At present, this family comprises two subfamilies, Pantherinae and Felinae, 14 genera and 46 species (Mammal Diversity Database, 2026; https://www.mammaldiversity.org/). Felids are widely distributed across all continents except Antarctica, ranging from the frigid northern reaches of Russia in the north to the hot, arid southern tip of Africa in the south, and occupy a broad spectrum of habitat types (Kamrava et al. 2024). Felids display highly conserved phenotypic traits. For example, they possess markedly shortened rostrums and exceptional night vision. They are agile and adept at leaping, high‐speed running, and sharp turning maneuvers. With the exception of cheetahs ( *Acinonyx jubatus* ), all Felidae species retain retractable claws (Sunquist and Sunquist 2002; Bryant et al. 1996). However, there is a vast difference in body size among different felid species. The rusty‐spotted cat ( *Prionailurus rubiginosus* ) and the black‐footed cat ( *Felis nigripes* ), which constitute the smallest Felidae, have a body weight of just 1 to 2 kg; in contrast, some subspecies of the largest felid, the tiger ( *Panthera tigris* ), can weigh nearly 300 kg—a difference of more than 200 times (Lamberski 2014). With their extraordinary variation in body size, felids represent a highly suitable taxonomic group for investigating body size evolution across mammalian lineages.

Advances in theory and analytical techniques now allow body size trends within lineages to be reconstructed with considerable accuracy. A previous study of felid phylogeny found evidence of two optimal body size values, corresponding to large and small body sizes, respectively. Lineages evolving toward the larger optimum included species of the genus *Panthera*, the cheetah, the puma ( *Puma concolor* ), and all extinct felids. However, this conclusion is sensitive to several factors, such as whether extinct felids are included, the choice of algorithms, and the temporal selection of fossil taxa (Cuff et al. 2015). Advances in the quantity and quality of publicly available genomic resources have now made it possible to elucidate the molecular mechanisms underlying biological phenotypes. Numerous genomics‐based studies investigating the molecular basis of body size variation in vertebrates have yielded significant results, spanning a wide range of taxonomic groups, including Carnivora, Cetacea, Primates, and Serpentes (Xia, Zhou, et al. 2025; Harcourt and Schreier 2009; Sun et al. 2019; Xia, Sun, et al. 2025; Huang et al. 2021). However, research on the molecular basis of body size variation within Felidae remains limited.

Using 17 high‐quality Felidae genomes currently available, we carried out a comparative genomic analysis. First, we analyzed positively selected genes (PSGs) and rapidly evolving genes (REGs) in two large‐bodied Felidae species represented in this dataset. Second, we used phylogenetic generalized least squares (PGLS) methods to identify body‐size‐associated genes (BAGs). It is anticipated that our findings will advance current understanding of the molecular mechanisms underpinning body size evolution in the Felidae.

## 2 Methods

### 2.1 Genomic and Phenotype Data Collection

In this study, we retrieved Felidae genome sequences from the NCBI database. We ultimately selected 17 high‐quality Felidae genomes with a high degree of completeness, along with one outgroup species—the house mouse ( *Mus musculus* ; GenBank accession number: GCA_000001635.9)—for subsequent analysis. Body weight data for the Felidae species included in this study were sourced from the PanTHERIA database (Jones et al. 2009). For Felidae species lacking body weight data in the PanTHERIA database, we adopted the mean adult mass values reported in *Fowler's Zoo and Wild Animal Medicine* (Miller and Fowler 2014). To facilitate subsequent analyzes, we categorized the selected Felidae species into three body size groups: small‐bodied (< 5 kg), large‐bodied (> 150 kg), and medium‐bodied (the remaining species). In defining these body size groupings, we considered not only the upper and lower limits of recorded body weight for each felid species, but also prey body size as a key ecological criterion. Detailed body weight data and corresponding dietary references for each species are provided in Table S1 (Xiong et al. 2016; Bisceglia et al. 2008; Martínez‐Calderas and Colima 2026; McLean et al. 2005; Tófoli et al. 2009; Karki et al. 2024; Mishra et al. 2025; Lavoie et al. 2019). Body weight data and genomic information for the 17 Felidae species included in the final analysis are summarized in Table 1.

**TABLE 1:** Body weight values and genomic details of the Felidae species used in this study.

Scientific name	Accession number	Scaffold N50 (Mb)	Mass (kg)	Body size	BUSCO (%)
*Felis nigripes*	GCA_032458615.1	146.9	1.36	Small‐bodied	86.6
*Prionailurus bengalensis*	GCA_016509475.2	148.6	2.79	Small‐bodied	98.9
*Felis catus*	GCA_018350175.1	148.5	2.88	Small‐bodied	98.8
*Leopardus geoffroyi*	GCA_018350155.1	152.6	3.50	Small‐bodied	98.8
*Lynx rufus*	GCA_022079265.1	142.1	6.39	Medium‐bodied	98.6
*Herpailurus yagouaroundi*	GCA_014898765.1	49.3	6.75	Medium‐bodied	97.6
*Felis chaus*	GCA_019924945.1	148.6	7.17	Medium‐bodied	85.9
*Prionailurus viverrinus*	GCA_022837055.1	144.9	8.83	Medium‐bodied	99.0
*Lynx canadensis*	GCA_007474595.2	147.3	9.77	Medium‐bodied	95.8
*Neofelis nebulosa*	GCA_028018385.1	150.2	15.10	Medium‐bodied	98.8
*Panthera uncia*	GCA_023721935.1	112.1	50.00	Medium‐bodied	96.9
*Acinonyx jubatus*	GCA_027475565.2	144.4	50.50	Medium‐bodied	98.8
*Panthera pardus*	GCA_024362965.1	126.8	52.40	Medium‐bodied	98.9
*Puma concolor*	GCA_003327715.1	100.5	53.90	Medium‐bodied	85.2
*Panthera onca*	GCA_046562875.2	148.3	84.90	Medium‐bodied	95.9
*Panthera leo*	GCA_018350215.1	147.4	159.00	Large‐bodied	96.7
*Panthera tigris*	GCA_018350195.2	146.9	163.00	Large‐bodied	98.8

### 2.2 Genome Annotation

Among the 17 Felidae genomes selected for this study, two species ( *F. nigripes* and *Felis chaus* ) had only assembled genome sequences and lacked corresponding annotation information. To enable subsequent analyzes, we performed genome annotation for these two species.

First, *de novo* repeat libraries were built separately for each species by applying RepeatModeler v2.0.1 (Flynn et al. 2020). Following this, RepeatMasker v4.1.0 (Chen 2004) was used to detect both known and novel transposable elements (TEs), which involved aligning genomic sequences against the species‐specific *de novo* repeat libraries as well as the Repbase database. Our protein‐coding gene prediction pipeline integrated *de novo* prediction with homology‐based evidence. For *de novo* gene prediction, we utilized three software tools—Augustus v2.5.5 (Stanke and Waack 2003), GlimmerHMM v3.0.4 (Majoros et al. 2004), and Geneid v1.4.4 (Parra et al. 2000)—with their internal gene models to predict gene structures. For homology‐based prediction, protein sequences from four species ( *Homo sapiens* , *Felis catus* , *Canis lupus familiaris* , and *M. musculus* ) were retrieved as templates; these sequences were then aligned to the assembled genomes using Miniprot v0.12 (Li 2023). Subsequently, EVidenceModeler v2.1.0 (Haas et al. 2008) was employed to integrate the *de novo* and homology‐predicted genes, thereby constructing non‐redundant gene sets. From these non‐redundant gene sets, short genes (< 50 amino acids) and prematurely terminated genes were excluded to generate the final complete gene sets. The quality of the final annotation results was assessed using BUSCO v5.2.2 (Simão et al. 2015) with the “mammalia_odb10” database.

### 2.3 Screening of Orthologous Gene and Reconstruction of Phylogenetic Tree

To clarify phylogenetic relationships within Felidae, we first assembled a genomic dataset and performed a phylogenetic analysis, upon which all downstream inferences were based. OrthoFinder v2.4.0 (Emms and Kelly 2015), coupled with the Diamond (Buchfink et al. 2015) algorithm, was used to detect all one‐to‐one orthologous genes among the sampled genomes. In this study, we adopted Diamond rather than BLAST to conduct all‐vs‐all protein sequence alignment. Relative to traditional BLAST, Diamond delivers a significant acceleration in runtime and greatly enhances computational efficiency without compromising alignment precision. Amino acid sequences of the single‐copy orthologs were aligned with MUSCLE v3.8.31 (Edgar 2004), and the resulting protein alignments were converted to codon alignments using the Perl script PAL2NAL (Suyama et al. 2006). A maximum likelihood tree was inferred from the concatenated single‐copy ortholog alignments in RAxML v8.1.12 (Stamatakis 2014) under the GTR‐GAMMA model, with the options ‐m GTRGAMMA ‐f a ‐x 12345 ‐N 1000 ‐p 12345 ‐T 64. Branch support was evaluated with 1000 rapid bootstrap replicates. Divergence times across Felidae were then estimated using MCMCTREE in PAML v4.10.7 (Yang 1997), applying ‐clock 2 ‐alpha 0.5 ‐model 3. The temporal scale was calibrated with minimum‐age constraints taken from the TimeTree (Kumar et al. 2017) (https://www.timetree.org) and grounded in fossil evidence: a minimum of 1.5 Mya for the split between *Panthera uncia* and *P. tigris* , a minimum of 3.8 Mya for the divergence between *Neofelis* and *Panthera*, and a minimum of 5.3 Mya for the separation of *Lynx* from the *Prionailurus* + *Felis* clade (Barnett et al. 2020).

### 2.4 Identification of BAGs Using PGLS

We performed PGLS analysis using the caper package in R (Orme et al. 2013) to examine the association between the evolutionary rates of individual genes and body weight as a phenotypic trait. The ultrametric phylogenetic tree of Felidae used for PGLS analysis in this study was obtained from the aforementioned phylogenetic inference. The body size phenotypic dataset consisted of body weight values collected following the protocols documented in previous sections. We estimated evolutionary rates (*ω*) using the free‐ratio model (model = 1) implemented in the codeml program of PAML v4.10.7. We then calculated the mean *ω* values across all branches from the ancestral node to the terminal tip for each Felidae species to obtain the root‐to‐tip evolutionary rate (*ω*). We performed the analysis under the Brownian motion model and estimated the phylogenetic signal parameter (*λ*) using maximum likelihood estimation. The parameter *λ* quantitatively characterizes the strength of the phylogenetic signal; values closer to 1 indicate a stronger phylogenetic effect (Pagel 1999). Finally, we subsequently calculated and assessed the correlation between log‐transformed root‐to‐tip *ω* values and log‐transformed raw body weight phenotypic values. Statistical significance was determined with a threshold of FDR‐corrected *p* < 0.05, for which multiple testing correction was conducted following the standard Benjamini‐Hochberg (BH) approach.

### 2.5 Selective Pressure Analysis

To investigate the effects of natural selection on body size‐related genes in Felidae, we performed selection pressure analysis using the codeml program implemented in PAML v4.10.7, with all one‐to‐one orthologous genes previously identified by OrthoFinder as input. The ratio of nonsynonymous to synonymous substitution rates (dN/dS), denoted *ω*, is a widely used metric for measuring natural selection pressure. An *ω* value greater than 1 indicates that beneficial nonsynonymous mutations are enriched by natural selection, with their fixation rate exceeding that of neutral synonymous mutations—the canonical signature of positive selection. Large‐bodied and small‐bodied Felidae were separately designated as the foreground branches, with the remaining branch as the background branch. We applied the branch‐site model (Ma: model = 2, NSsites = 2, fix_omega = 0; null model Ma0: model = 2, NSsites = 2, fix_omega = 1, omega = 1) to detect PSGs in each foreground group. Statistical significance was determined by likelihood ratio test (LRT) and chi‐square tests. Genes with an FDR‐corrected *p* < 0.05 and significantly deviating from the null model were considered PSGs. To minimize false positives, we further filtered the candidate genes using the following criteria: genes containing three or more consecutive positively selected sites were excluded; genes with positively selected sites located at the start or end of the coding region were excluded; and genes whose positively selected sites had a Bayes Empirical Bayes (BEB) posterior probability below 0.95 were excluded (Lyu et al. 2025). Additionally, we employed the branch model (M2: model = 2, NSsites = 0; null model M0: model = 0, NSsites = 0) to identify REGs in large‐bodied Felidae. When the evolutionary rate of a given gene is significantly higher in the foreground clade of large‐bodied Felidae relative to the background clade, the gene is defined as a REG shared across the large‐bodied Felidae clade. A likelihood ratio test was used to compare the two models, with a significance threshold set at an FDR‐corrected *p* < 0.05.

### 2.6 GO And KEGG Enrichment Analyzes

This study employed semantic similarity analysis of Gene Ontology (GO) terms and Kyoto Encyclopedia of Genes and Genomes (KEGG) pathways to identify biological processes and functional pathways associated with BAGs, PSGs, and REGs. Protein sequences were functionally annotated using InterProScan v5.16–93 (Jones et al. 2014) against the InterPro database, and then subjected to KEGG pathway annotation. Chi‐square tests were conducted for statistical significance analysis, and a *p*‐value below 0.05 was predefined as the significance criterion.

## 3 Results

### 3.1 Annotation Results for Two Felidae Species

In this study, we annotated the genome assemblies of *F. nigripes* and *F. chaus* ; the repeat content of the two genomes was 40.31% and 40.67%, respectively. Furthermore, we annotated approximately 16,640 and 16,577 protein‐coding genes in these two genomes, respectively. These results are largely consistent with genomic data from other closely related species of Felidae (Armstrong et al. 2022; Cho et al. 2013). We assessed the annotation quality of all Felidae genomes used in this study with BUSCO, employing the mammalia_odb10 database under default parameters. For all species, more than 85% of the genes were classified as complete. Detailed results are presented in Table 1. These findings indicate that the quality of the genome annotations in this study is sufficient for downstream analyzes.

### 3.2 Screening of Orthologous Gene and Reconstruction of Phylogenetic Tree

**FIGURE 1:**
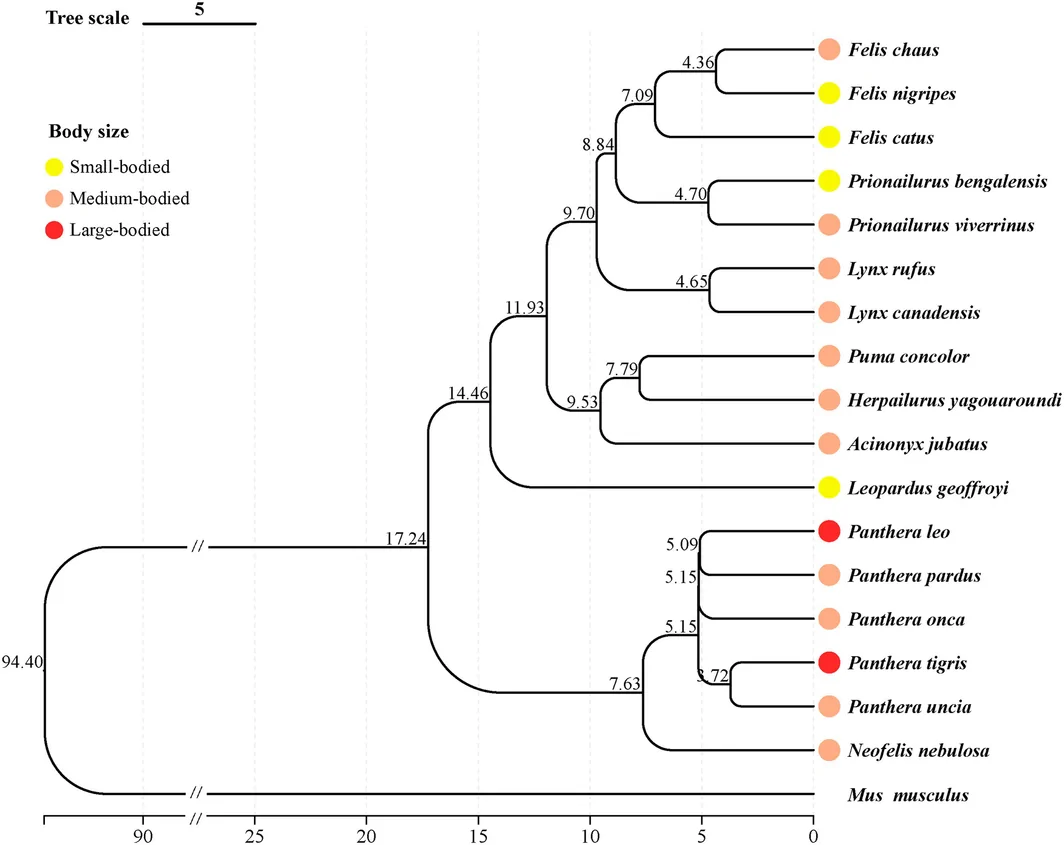
This phylogenetic tree depicts the body sizes of the Felidae species examined in this work. Circle colors correspond to different body size classes: Yellow for small‐bodied, orange for medium‐bodied, and red for large‐bodied species. Numbers above the tree nodes indicate speciation divergence times, in millions of years ago (Mya).

We applied OrthoFinder to 18 genomes and recovered 20,704 orthogroups in total. Across all species, 9162 orthogroups were shared, including 7875 that were strictly single‐copy. We aligned and concatenated these single‐copy genes and inferred the species phylogeny, thereby providing a framework for subsequent comparative genomics analyzes (Figure 1). The phylogenetic relationships inferred in this study are broadly consistent with those previously reconstructed from concatenated sequences of exons (Barnett et al. 2020).

### 3.3 Genome Scanning of BAGs

**FIGURE 2:**
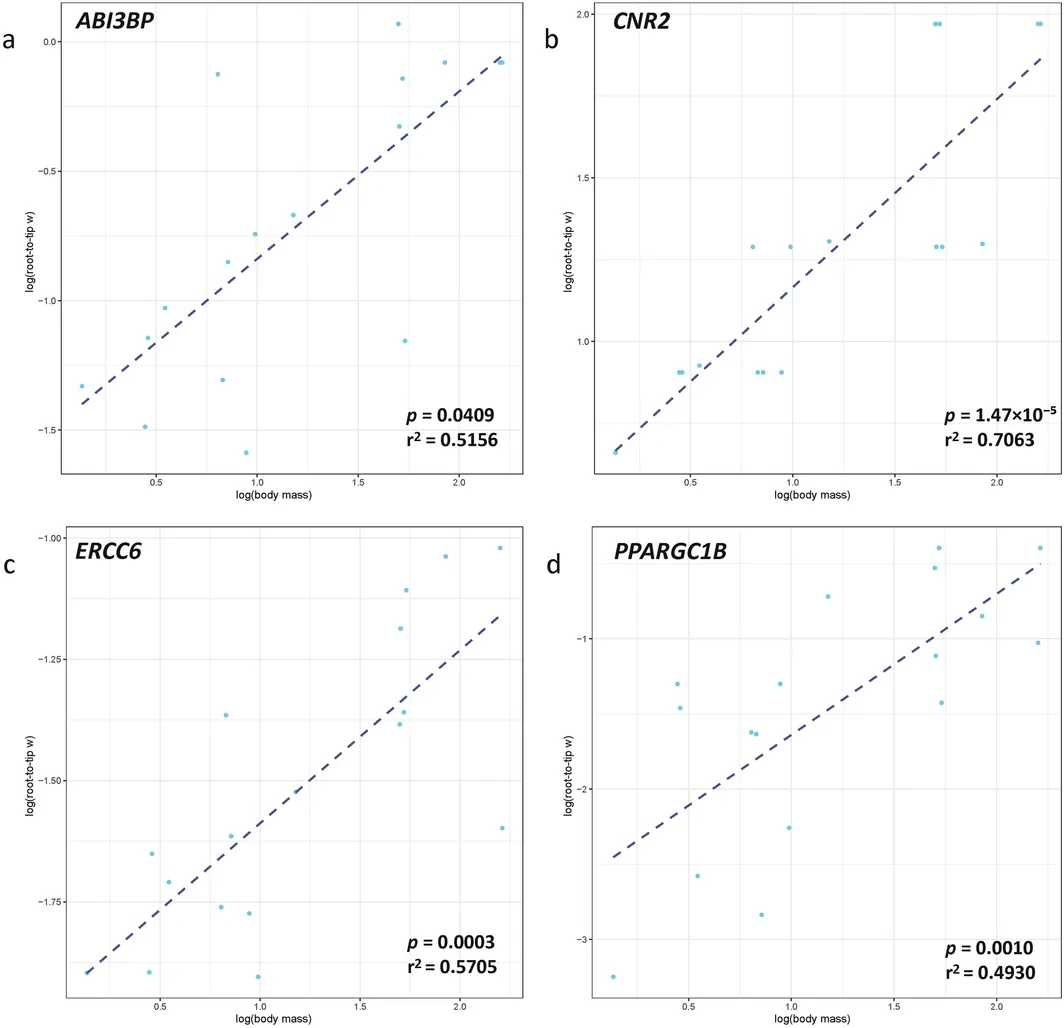
Regression analysis of root‐to‐tip ratios (*ω*) against body mass using PGLS in R for BAGs: *ABI3BP* (a), *CNR2* (b), *ERCC6* (c), *PPARGC1B* (d).

Using PGLS, we identified 35 genes significantly associated with body weight in Felidae species, with a significance threshold of FDR‐corrected *p* < 0.05. We formally defined these genes significantly associated with Felidae body weight as BAGs (Table S2). Additionally, we plotted the relationship between the coefficient of determination (*r* ^2^) and FDR‐corrected *p* (Figure S1). Notably, multiple BAGs identified in this study have been well documented to be involved in the regulation of mammalian body size‐related traits, including *ABI3BP*, *CNR2*, *ERCC6*, *HERC1*, *IL1A*, *PPARGC1B*, *SLC30A10*, and *TPO* (Figure 2). These genes have been functionally linked to organismal growth, skeletal system development, and lipid metabolism in mammals.

**FIGURE 3:**
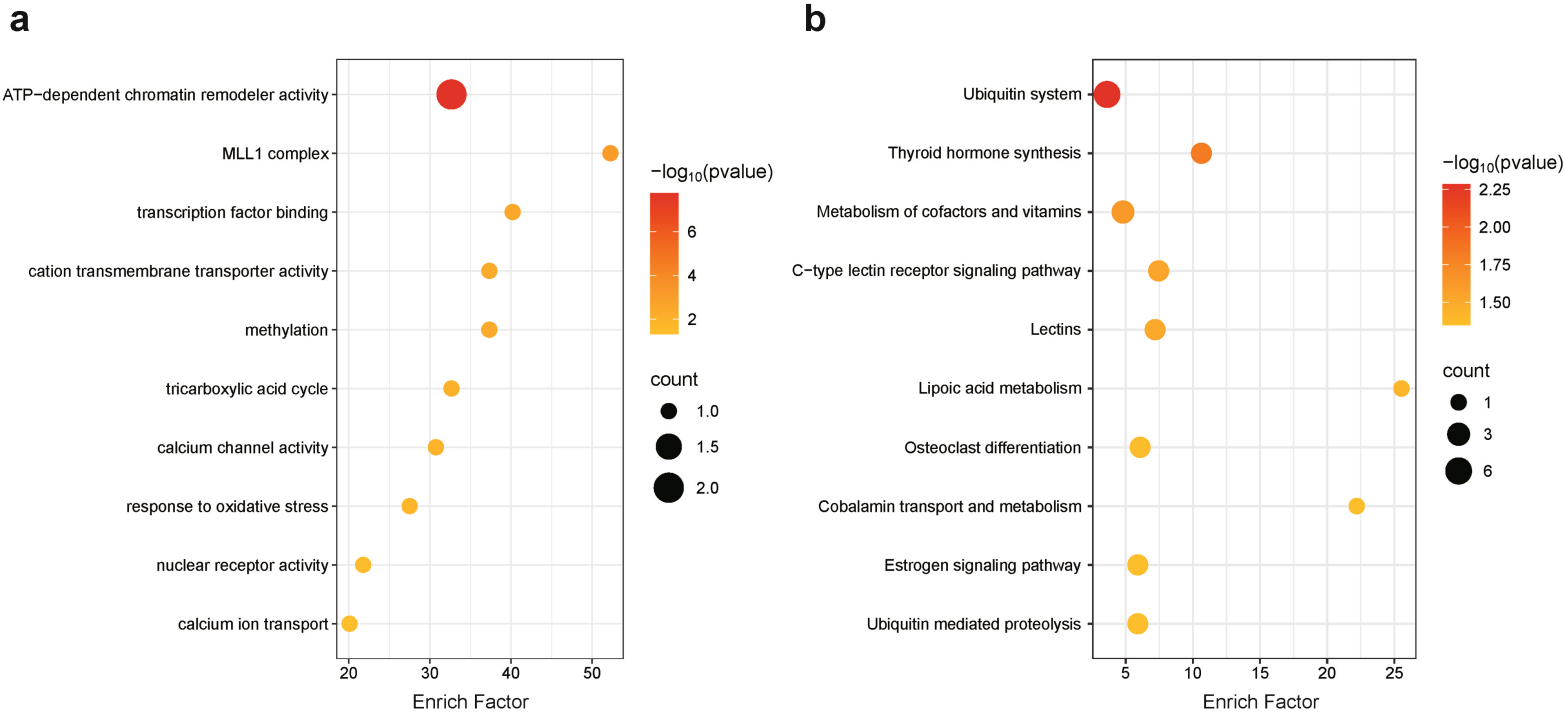
Functional enrichment visualization of BAGs: (a) GO term enrichment analysis plot of BAGs; (b) KEGG pathway enrichment analysis plot of BAGs.

We further performed GO enrichment analysis on these BAGs and found that they were significantly enriched in 18 GO terms (*p* < 0.05). These BAGs were significantly enriched in GO terms associated with growth and developmental regulation, including the MLL1 complex (GO:0071339), ATP‐dependent chromatin remodeler activity (GO:0140658), and methylation (GO:0032259). In addition, some of the enriched GO terms were associated with skeletal tissue development, including calcium channel activity (GO:0005262) and calcium ion transport (GO:0006816). Additionally, these BAGs were significantly enriched in 13 KEGG pathways, including osteoclast differentiation (ko04380), thyroid hormone synthesis (ko04918), and the estrogen signaling pathway (ko04915) (Figure 3 and Table S3).

### 3.4 Positive Selection Analyzes

**FIGURE 4:**
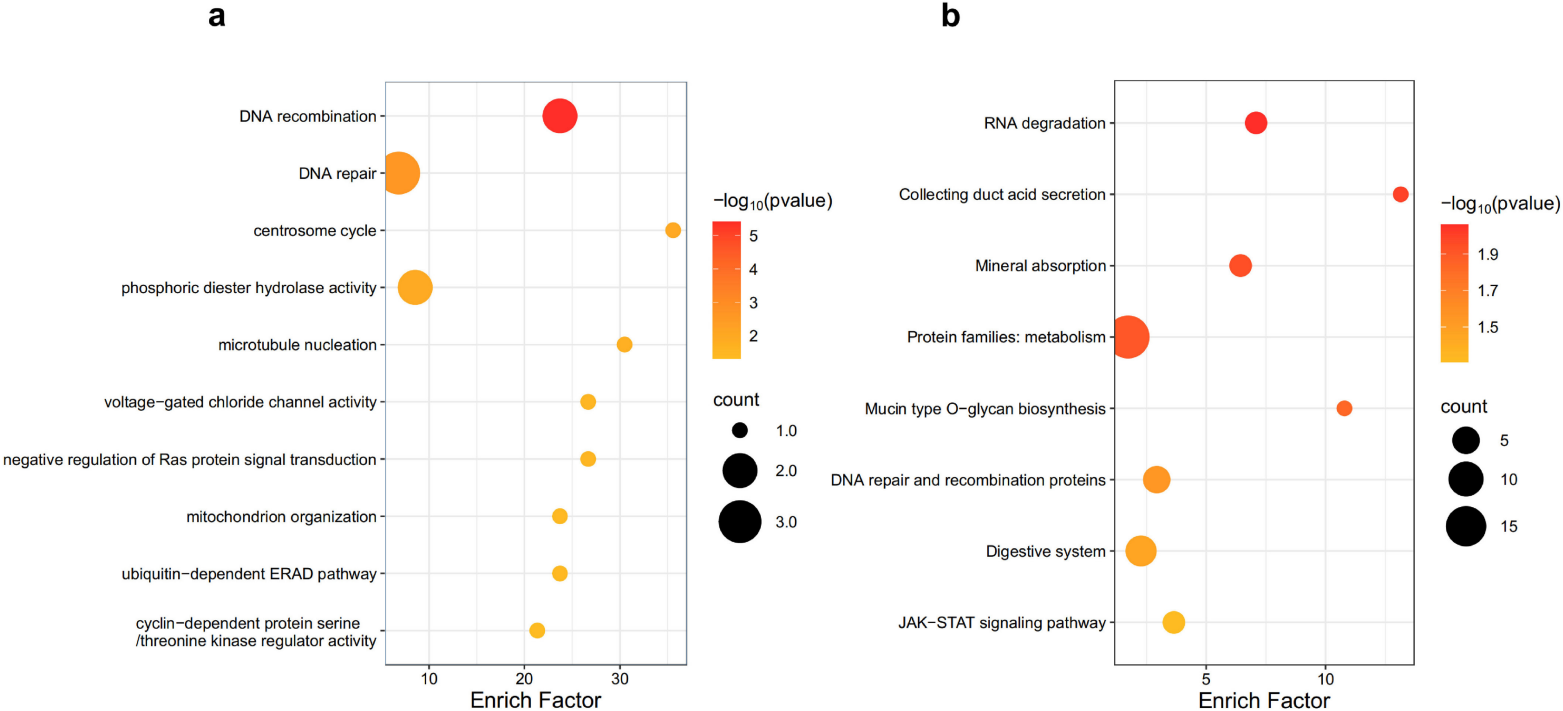
Enrichment plots of PSGs in large‐bodied Felidae species: (a) GO term enrichment analysis plot of PSGs; (b) KEGG pathway enrichment analysis plot of PSGs.

After screening and filtering, 92 PSGs were identified in the two large‐bodied Felidae species, *P. leo* and *P. tigris* . GO enrichment analysis revealed that these genes were significantly enriched in functions related to cell cycle regulation and cell proliferation, including DNA repair (GO:0006281), negative regulation of Ras protein signal transduction (GO:0046580), and cyclin‐dependent protein serine/threonine kinase regulator activity (GO:0016538). Consistently, KEGG enrichment analysis demonstrated that these PSGs were significantly enriched in several key biological pathways, including DNA repair and recombination proteins (ko03400), JAK–STAT signaling pathway (ko04630), mineral absorption (ko04978), and digestive system (ko09154) (Figure 4 and Table S4).

**FIGURE 5:**
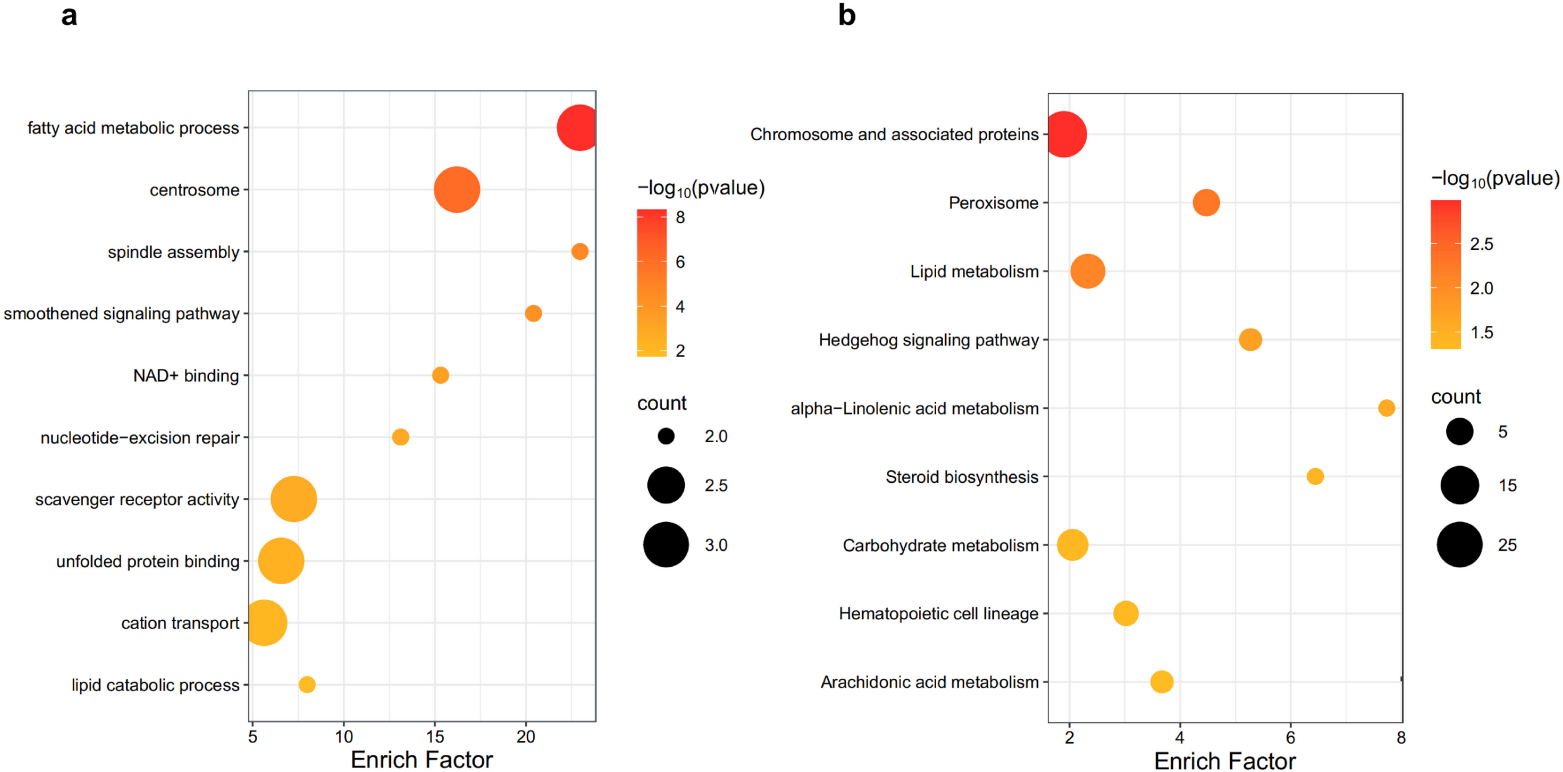
Enrichment plots of PSGs in small‐bodied Felidae species: (a) GO term enrichment analysis plot of PSGs; (b) KEGG pathway enrichment analysis plot of PSGs.

In contrast to the enrichment results observed in large‐bodied Felidae, we identified 207 PSGs across four small‐bodied Felidae species. GO enrichment analysis revealed that these PSGs were significantly enriched in GO terms related to lipid and energy metabolism, including fatty acid metabolic process (GO:0006631), lipid catabolic process (GO:0016042), NAD^+^ binding (GO:0070403), and smoothened signaling pathway (GO:0007224). The results of the KEGG enrichment analysis showed that these genes were significantly enriched in pathways including lipid metabolism (ko09103), steroid biosynthesis (ko00100), and Hedgehog signaling pathway (ko04340) (Figure 5 and Table S5).

### 3.5 Rapidly Evolving Genes Analyzes

Using the branch model in PAML, we identified 324 REGs in large‐bodied Felidae. The enrichment analysis results showed that these REGs were primarily enriched in functions and pathways including the G protein‐coupled receptor signaling pathway (GO:0007186), G protein‐coupled receptor activity (GO:0004930), and actin binding (GO:0003779) (Table S6).

## 4 Discussion

Dispersal and coevolution are considered the primary factors driving the speciation of modern Felidae over the past 10 million years (Johnson et al. 2006; Johnson and O'Brien 1997). Although Felidae share numerous common characteristics, they exhibit significant variation in body size. Previous studies have suggested that body size differences are typically closely related to life history, metabolic rate, population size, and various ecological factors (Gittleman 1985). Among closely related species, body size divergence is typically interpreted as an adaptive strategy to reduce interspecific competition via character displacement and trophic niche partitioning (Schluter 2000). For example, small‐bodied Felidae typically prey on vertebrates smaller than themselves, while large‐bodied Felidae tend to feed on large ungulates of comparable size. This difference allows them to share habitat resources and reduce direct competition (Donadio and Buskirk 2006). In a recent study based on the Felidae fossil record and utilizing a Bayesian analytical framework, two coupled independent increases in body size were identified in Felidae evolutionary history: first, a gradual increase in body size within the subfamily Machairodontinae over the past 15 million years; second, a rapid increase in body size among members of *Panthera* over the past 5 million years. In addition, some groups, such as the genera *Leopardus*, *Lynx*, and *Felis*, exhibited an evolutionary trend of gradual reduction in body size. The study suggests that major historical environmental changes were key factors influencing the evolutionary trajectory of Felidae, driving their body size evolution toward forms adapted for hunting large herbivores (Porto et al. 2025). Existing research has comprehensively characterized body size variation in Felidae at both macroscopic and molecular scales, yet the genetic underpinnings of extreme body size differentiation across Felidae still lack systematic in‐depth investigation. Using 17 high‐quality Felidae genomes, this study employed comparative genomics and bioinformatics methods to systematically investigate the molecular adaptive mechanisms underlying Felidae body size evolution. At the molecular level, this study confirms that body size‐associated genes have been subject to distinct evolutionary selective pressures across Felidae species, providing a novel basis for elucidating the molecular mechanisms of Felidae body size evolution.

Body size is a complex multidimensional continuous trait, orchestrated by the coordinated interactions of a large set of genes, gene families, and signaling pathways (Xia, Sun, et al. 2025). *Hox* genes represent a class of highly conserved transcription factor‐encoding genes in animals, acting as master regulators that govern growth and developmental processes including organ morphogenesis, as well as skeletal and limb growth and patterning (Soshnikova and Duboule 2008; Pineault et al. 2015). MLL1 is a histone methyltransferase whose catalytic activity modulates *Hox* expression (Milne et al. 2002). Genetic knockout of *MLL* in mice disrupts normal *Hox* expression, leading to severe developmental abnormalities and lethality; targeted deletion of its methyltransferase domain further results in pronounced skeletal developmental defects (Yu et al. 1998; Terranova et al. 2006). In the present study, BAGs were significantly enriched in the MLL1 complex (GO:0071339), suggesting that this regulatory pathway plays a critical role in body size determination and skeletal development in Felidae. The skeleton constitutes the central structural framework of the animal body, and the dynamic balance between bone formation and remodeling dictates longitudinal bone length and overall skeletal size. In vertebrates, bone mass and skeletal phenotype are maintained by the functional equilibrium between osteoclast and osteoblast activity (Ofek et al. 2006). Consistent with this, the BAGs identified in this study were significantly enriched in bone‐associated pathways, including osteoclast differentiation (ko04380), calcium channel activity (GO:0005262), and calcium ion transport (GO:0006816). BAGs were also enriched in thyroid hormone synthesis (ko04918). Thyroid hormones are essential for regulating basal metabolism, bone development, and growth. In studies of body size variation among macaque species, this pathway was significantly enriched and is thought to be closely associated with body size and growth (Wang et al. 2024). Collectively, these findings suggest that skeletal development and its regulation may represent core factors driving body size divergence and evolution in Felidae.

Beyond the pathway‐level adaptive evolution described above, this study further suggests that multiple individual genes within the identified BAGs set may also contribute to the regulation of body size in Felidae. The *ERCC6* gene encodes a DNA/RNA helicase involved in the nucleotide excision repair pathway (Troelstra et al. 1992). Previous studies have linked mutations in this gene to Cockayne syndrome, a human genetic disorder characterized by postnatal growth retardation, developmental delay, and skeletal abnormalities. Mice with *ERCC6* knockout exhibit symptoms resembling those of human Cockayne syndrome; their body weight decreases significantly after birth compared with wild‐type mice, indicating that growth and development are affected to some extent (van der Horst et al. 1997). Similarly, an association analysis of Kashin‐Beck disease based on CNV data from whole‐genome resequencing showed that *ABI3BP* is overexpressed in patients and is considered significantly associated with the disease. Kashin‐Beck disease is a form of chondrodysplasia that typically manifests in early childhood, with symptoms such as growth retardation, abnormal joint development, and short stature (Zhang et al. 2014). Ubiquitin ligases may play a key role in regulating bone metabolism, and *HERC1* encodes one such ligase. In mice with *HERC1* knockout, an imbalance between bone growth and resorption was observed relative to wild‐type mice, resulting in reduced bone mass. Studies have shown that *HERC1* regulates cells during bone growth and thereby maintains bone homeostasis; it is also considered a potential candidate gene for human bone‐related diseases (Pedrazza et al. 2023).

In addition, *CNR2* (cannabinoid receptor Type 2) knockout mice also exhibit decreased bone mass and bone mineral density, demonstrating that this gene exerts critical regulatory functions in skeletal homeostasis and offering novel therapeutic implications for osteoporosis, a prevalent human skeletal disorder (Ofek et al. 2006; Karsak et al. 2005). Other studies have linked *CNR2* to body weight regulation: in mice, brain expression of *CNR2* leads to weight loss, whereas in humans, resequencing‐based genotyping suggests associations with obesity‐related factors such as insulin and leptin levels (de Luis et al. 2017; Romero‐Zerbo et al. 2012). Using the same approach, *IL1A* was identified as associated with body weight in obese individuals. Furthermore, *IL1A* overexpression was detected in mice that became obese on a high‐fat diet, supporting the notion that this gene may play an important regulatory role in obesity and fat metabolism (Um et al. 2011). An association analysis of the *PPARGC1B* gene in Hu sheep showed that it is significantly associated with body weight, making this gene an important candidate gene for economic traits (Zhang et al. 2022). Finally, we identified two genes among the BAGs that are closely associated with thyroid hormones. SLC30A10 is a cell‐surface transporter involved in manganese transport. In *SLC30A10* knockout mice, thyroid function was impaired relative to wild‐type mice, thyroid hormone levels plummeted, and manganese levels surged dramatically in multiple tissues. Phenotypically, mutant mice showed reduced stature and slow or halted weight gain (Hutchens et al. 2017). The *TPO* gene encodes thyroid peroxidase, an enzyme involved in thyroid hormone synthesis. Mice carrying missense mutations in *TPO* exhibit markedly stunted growth and excessive thyroid enlargement. These mice have reduced thyroid hormone levels, resembling human congenital hypothyroidism, a condition characterized by early childhood growth and developmental delays (Takabayashi et al. 2006). Collectively, the BAGs identified in this study are functionally enriched in core biological processes tightly linked to body size determination, including growth and development, skeletal development, and lipid metabolism regulation. Previous comparative studies on body size evolution across Carnivora have demonstrated that body size‐associated genes in this order are predominantly associated with obesity and lipid metabolism (Huang et al. 2021). In contrast, our findings reveal that BAGs in Felidae bear a stronger functional association with skeletal development and thyroid hormone homeostasis. These results further support the notion that animal body size is not governed by a small set of individual genes. Instead, body size regulation relies on the coordinated interplay of broad‐scale signaling pathways and the precise fine‐tuning of genes underlying core biological processes across multiple physiological systems.

PSGs identified in large‐bodied Felidae are significantly enriched in DNA repair (GO:0006281), and DNA repair and recombination proteins (ko03400). Multicellular organisms undergo continuous cell division, and errors inevitably occur during this process with a certain probability. Theoretically, larger and longer‐lived species, which undergo more cell divisions over their lifetimes, should have a higher incidence of cancer than smaller and shorter‐lived species. Despite the intuitive expectation, cross‐species comparisons have failed to reveal a relationship between cancer prevalence and body size, a phenomenon termed Peto's paradox (Peto 2016). Although Peto's paradox is currently the subject of renewed debate, the academic community generally agrees that large, long‐lived species have evolved a variety of distinct tumor‐suppressing mechanisms (Butler et al. 2025). Large animals (such as African elephants) possess more efficient DNA repair systems, an increased number of tumor suppressor genes, and other potential mechanisms (Abegglen et al. 2015). Genomic studies on body size evolution in Carnivora have identified multiple BAGs associated with DNA repair and cancer (Huang et al. 2021). Combining these findings with the results of this study, this study supposes that large‐bodied Felidae can reduce the accumulation of mutated cells through enhanced DNA repair capabilities and eliminate cells with DNA damage, thereby achieving a reduced risk of cancer. These findings provide a novel molecular basis for Peto's paradox in Felidae. The Hippo signaling pathway is a highly evolutionarily conserved kinase cascade. Extensive studies in model organisms have demonstrated that it plays a central role in determining organ and organismal body size by precisely balancing cell proliferation and apoptosis (Yu et al. 2015). The REGs identified in the large‐bodied Felidae clade in our study were significantly enriched in the G protein‐coupled receptor signaling pathway (GO:0007186) and G protein‐coupled receptor activity (GO:0004930). Multiple studies have demonstrated that the Hippo pathway is regulated by various upstream mechanisms, including G protein‐coupled receptor (GPCR) signaling pathways (Miller et al. 2012; Yu, Mo, and Guan 2012). YAP and TAZ are homologous oncogenic transcriptional co‐activators involved in lysophosphatidic acid (LPA)‐induced gene expression, cell migration, and proliferation; they are also the major effectors of the Hippo pathway. GPCR signaling can effectively modulate the phosphorylation status and activity of YAP and TAZ depending on the type of G protein to which they are coupled, thereby activating or inhibiting the Hippo‐YAP pathway (Yu, Zhao, et al. 2012; Luo and Yu 2019). Therefore, we propose that this pathway also plays a significant role in the adaptive evolution of body size in Felidae. Basal metabolic rate, defined as oxygen consumption per unit body mass, exhibits an allometric relationship with the 0.75th power of body weight. In other words, small‐bodied mammals have a higher metabolic rate per unit body weight, a characteristic that necessitates the consumption of foods with higher nutritional value and energy density (Gittleman 1985). The PSGs identified in this study in small‐bodied Felidae were significantly enriched in pathways related to energy and lipid metabolism, including NAD^+^ binding (GO:0070403), lipid catabolic processes (GO:0016042), and lipid metabolism (ko09103). This finding is consistent with the high energy requirements of small‐bodied mammals. Furthermore, these PSGs are also enriched in the Hedgehog signaling pathway (ko04340), which, like the Hippo signaling pathway, is involved in regulating cell proliferation, growth, and body size in vertebrates (Vukicevic and Sampath 2012).

Lastly, several limitations of the present study should be recognized. Owing to the currently limited availability and uneven quality of Felidae genomic resources, our analyzes were restricted to high‐quality genomes from only 17 felid species. Specifically, only two large‐bodied felid species—the tiger and the lion—were represented in the large‐sized clade. Although body weight was treated as a continuous trait in our PGLS analysis, and the body size grouping was established by comprehensively integrating prey size, ecological niche, and the actual upper and lower limits of body weight, we acknowledge that this grouping scheme retains an inherent degree of subjectivity. Looking forward, the continued release and curation of high‐quality Felidae genomes and their annotations will expand taxonomic coverage in future analyzes, enabling more fine‐grained and comprehensive investigations into body size variation and evolution across the family. Furthermore, several of the BAGs identified in this study lack prior functional characterization, including validation via cellular experiments or organismal gene editing studies. These candidate genes therefore provide valuable foundational resources and novel research directions for future explorations of the molecular mechanisms underlying mammalian body size evolution.

## 5 Conclusions

This study performed reannotation of the genomes of two felid species and conducted the first genomic analysis of body size variation and evolution in Felidae. Our results demonstrate that felid body size evolution is driven by adaptive changes in both core signaling pathways and individual key genes. Comparative genomic analyzes demonstrate that body size evolution in Felidae is tightly linked to skeletal development, lipid regulation, and growth hormone modulation. At the pathway level, this association is reflected in enriched functional categories including the MLL1 complex and thyroid hormone synthesis. At the gene level, we identified multiple BAGs, including *ABI3BP*, *CNR2*, *ERCC6*, *PPARGC1B*, and *SLC30A10*. Furthermore, a subset of our findings is consistent with conclusions from body size evolution studies in other mammals, suggesting that molecular mechanisms regulating body size may share commonalities across mammalian lineages. This provides a reference for future efforts to elucidate the molecular mechanisms underlying the complex and diverse phenotypic variations in mammals.

## Author Contributions

**Shihu Zhao:** conceptualization (lead), data curation (lead), formal analysis (equal), investigation (lead), methodology (lead), software (equal), visualization (lead), writing – original draft (lead), writing – review and editing (equal). **Tian Xia:** data curation (equal), formal analysis (equal), methodology (equal), software (equal), writing – review and editing (equal). **Xuesong Mei:** data curation (equal), formal analysis (equal), methodology (equal), software (equal), writing – review and editing (equal). **Chen Qiu:** formal analysis (equal), software (equal), writing – review and editing (equal). **Ying Zhang:** formal analysis (equal), software (equal), writing – review and editing (equal). **Chao Zhao:** funding acquisition (equal), methodology (equal), software (equal). **Xiaoyang Wu:** data curation (equal), funding acquisition (equal), visualization (equal). **Xiaodong Gao:** data curation (equal), funding acquisition (equal), visualization (equal). **Jiaohui Fang:** data curation (equal), funding acquisition (equal), visualization (equal). **Jianqun Ding:** data curation (equal), visualization (equal). **Zhenglong Wang:** data curation (equal), visualization (equal). **Xiufeng Yang:** project administration (equal), supervision (equal), writing – review and editing (equal). **Honghai Zhang:** funding acquisition (lead), project administration (lead), writing – review and editing (equal).

## Funding

This work was supported by the National Natural Science Foundation of China (Grants 32301319, 32470436, and 32470448), Postdoctoral Innovation Project of Shandong Province (Grant SDCX‐ZG‐202400113), Postdoctoral Fellowship Program of CPSF (Grant GZC20240887), and Youth Innovation Team in Colleges and Universities of Shandong Province (Grant 2022KJ177).

## Ethics Statement

The authors have nothing to report.

## Consent

The authors have nothing to report.

## Conflicts of Interest

The authors declare no conflicts of interest.

## Supporting information


**Figure S1:** The relationship between the coefficient of determination (*r* ^2^) and FDR‐corrected *p*‐values. The *x*‐axis represents FDR‐corrected *p*‐values, and the *y*‐axis represents adjusted *r* ^2^ values. The red dashed line marks the *p* = 0.05 significance threshold. A consistent trend is observed: genes with smaller *p*‐values generally correspond to higher *r* ^2^ values.

**Table S1:** Body mass data and literature summary for all included Felidae species.

**Table S2:** Body size‐associated genes (BAGs) identified in this study.

**Table S3:** GO and KEGG enrichment results of body size‐associated genes (BAGs).

**Table S4:** Positively selected genes (PSGs) in large‐bodied Felidae and their GO and KEGG enrichment results.

**Table S5:** Positively selected genes (PSGs) in small‐bodied Felidae and their GO and KEGG enrichment results.

**Table S6:** Rapidly evolving genes (REGs) in large‐bodied Felidae and their GO enrichment results.

## Data Availability

All genome sequences used in this study are available on the NCBI database (https://www.ncbi.nlm.nih.gov) under the accession numbers reported in Table 1.
